# *QuickStats:* Age-Adjusted Suicide Rates,* by Method of Suicide^†^ — National Vital Statistics System, United States, 2001–2021

**DOI:** 10.15585/mmwr.mm7237a4

**Published:** 2023-09-15

**Figure:**
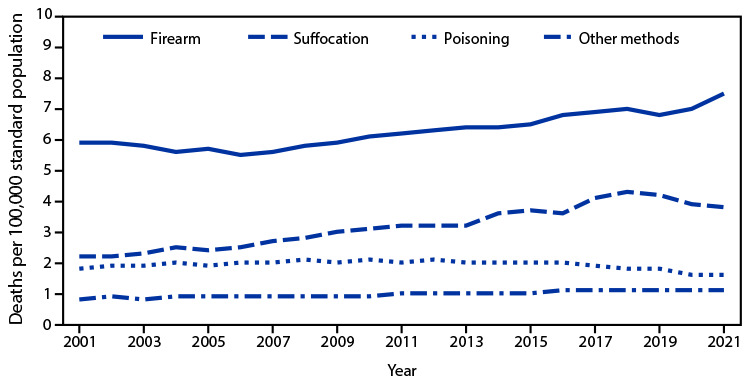
During 2001–2021, age-adjusted suicide rates involving firearms, suffocation, and other methods generally increased, and those involving poisoning decreased. Rates of firearm-related suicide were stable from 2001 (5.9 deaths per 100,000 population) to 2006 (5.5), and then increased through 2021 (7.5). Rates of suffocation-related suicide increased from 2.2 deaths in 2001 to 4.3 in 2018, and then decreased slightly through 2021 (3.8). After a period of increasing and then stable rates during 2001–2016, suicide rates attributed to poisoning decreased from 2.0 in 2016 to 1.6 in 2021. Firearm-related suicide had the highest rates during the period.

**Source:** National Center for Health Statistics, National Vital Statistics System, Mortality Data, 2001–2021. https://www.cdc.gov/nchs/nvss/deaths.htm

For more information on this topic, CDC recommends the following link: https://www.cdc.gov/suicide

